# Patient safety in home health care: a grounded theory study

**DOI:** 10.1186/s12913-023-09458-9

**Published:** 2023-05-10

**Authors:** Sahar Keyvanloo Shahrestanaki, Forough Rafii, Tahereh Najafi Ghezeljeh, Mansoureh Ashghali Farahani, Zahra Amrollah Majdabadi Kohne

**Affiliations:** 1 School of Nursing and Midwifery, Iran University of Medical Sciences, Tehran, Iran; 2 Nursing and Midwifery Care Research Center, School of Nursing and Midwifery, Iran University of Medical Sciences, Tehran, Iran; 3 Cardiovascular Nursing Research Center, Rajaie Cardiovascular Medical and Research Center, Iran University of Medical Sciences, Tehran, Iran

**Keywords:** Safe care, Home care, Home health care, Grounded theory

## Abstract

**Background:**

The home environment is designed for living, not for professional care. For this reason, safe patient care is one of the most important challenges of home health care. Despite abundant research on safe care, there is still little understanding of safety issues in home care.

**Design:**

The aim of the present study was to explain the process of safe patient care in home health care. A qualitative, grounded theory study was conducted based on the approach proposed by Corbin & Strauss in 2015.

**Method:**

In total, 22 interviews were conducted with 16 participants including 9 home care nurses, 2 home care nursing assistants, 1 home care inspector, 1 home care physician and 3 family caregivers in Tehran, Iran. Four observation sessions were conducted in different homes. Purposeful sampling was used followed by theoretical sampling from August 2020-July 2022. Data analysis was carried out based on the approach proposed by Corbin & Strauss in 2015.

**Results:**

The results showed that the healthcare members (nurses, family caregivers, patients and home care centers) used the model of safe patient care in home health care based on four assessment methods, i.e. prevention, foresight, establishment of safety and verification. The core variable in this process is foresight-based care.

**Conclusion:**

The results of this study showed that the key to safe patient care in home health care, which helps to maintain patient safety and prevent threats to safe care, is the foresight of healthcare members, which is essential for identifying threats to safe care considering the many risks of home health care.

## Introduction

Today, the demand for home care has increased in the world [1, 2]. Home care services impose lower costs on patients and families, especially in case of patients with chronic diseases or older adults, compared to hospital services. Moreover, home care seems to be an important strategy for caring for these patients [3, 4]. Iran is no exception to this rule and has made a significant progress in this field in recent years [5]. One of the benefits of home care is that patients feel more comfortable in receiving care services [6]. However, an important issue is that the home environment is designed for living, not for caring. For this reason, safe patient care is one of the most important challenges of home care [7, 8].

## Background

Providing safe home health care depends on several factors [9]. One of these factors is the human factor, which relates to home care providers such as nurses [10]. Home care providers face many challenges that threaten patient safety, such as working with equipment, entering an unfamiliar environment, absence of other colleagues and the inability to receive advice from them, and communication problems with family members [11, 12].

Other healthcare members include family caregivers that are involved in round-the-clock home patient care and experience many problems such as fatigue from constant care, presence of home care nurses, and paying for treatment costs [13]. In addition to the human factor, environmental factors and risks such as the inappropriate infrastructure of the home environment, limited physical space, and inadequate room for equipment, devices [14] and drugs [15] also threaten safe patient care [16]. Finally, it can be stated that the safe patient care process depends on many factors. For this reason, it is necessary to investigate this issue from different aspects in order to provide safe care.

Despite the many risks of safe home care, there are no accurate statistics on the subsequent adverse complications [17, 18]. A review of the literature showed that most studies dealt with hospital safe care [19, 20] and little attention has been paid to safe home care [21, 22]. On the other hand, some studies only addressed one dimension of patient safety in home health care, such as human factors, and did not investigate other dimensions of safe [23]. In addition, a number of recent studies discussed the need for more studies in the field of patient safety in home care, especially in elderly and chronic patients [24–26]. In some other studies in this field, only the experiences of people involved in home care were considered and the issue of safe care was not investigated [27]. Nonetheless, none of these studies comprehensively addressed the model of safe patient care at home with a focus on safe care. In this regard, few studies have evaluated safe home care for patients with chronic diseases and older adults, which has led to numerous home health care challenges for patients and their caregivers [28].

Therefore, considering the research gap in this regard and the complex process of safe home care, the researchers decided to determine the model of patient safety in home health care, especially for patients with chronic diseases and older adults [29]. On the other hand, since one of the main goals of home care is to maintain patient safety [30] and this care is provided by care providers and family caregivers in an environment that is not designed for care [31], it is very important to investigate the determinants of patient safety in home health care from different aspects. Considering the above and the researchers' observation of some unwanted home care complications, the researchers tried to find out the interactions that occur between the safe health care members, the strategies these interactions lead to, and the models they create. To answer these questions and considering the importance of patient safety in home health care, the present study was conducted to explain the process of patient safety in home health care with a focus on chronic patients and older adults.

## Method

A qualitative study was carried out based on the grounded theory methodology [32] to explain the model of patient safety in home health care. In the present study, the grounded theory was a suitable methodology to describe the stages of experience and identification of the patient safety model in home health care, which is a phenomenon affected by social interactions between the patient, family, and health care providers [33].

## Participants

Nurses with at least two years of experience in home health care were included in the study. Family caregivers who took on the main burden of home care and lived with the patients were also included in the study. The inclusion criteria for patients were age 60 years and over and suffering from a chronic disease. All the participants understood and spoke Persian and were willing to participate in the study. Purposeful sampling was done followed by theoretical sampling until data saturation was reached from August 2020 to July 2022. The researcher tried to include participants with maximum variation in terms of age, gender, marital status, education level, and history of participation in home care [34]. Of 16 participants that were interviewed, 6 were interviewed twice (supplementary interviews). In this regard, face-to-face supplementary interviews were conducted with participants A, B, C, D, H, and N. A total of 22 interviews were conducted with 16 participants. Sixteen interviews were conducted with 9 home care nurses, 2 home nursing assistants, 1 home care inspector, 1 home care physician and 3 family caregivers of the patients. Four observation sessions were held in different homes. The participants’ characteristics are presented in Table 1.

**Table 1:** Characteristics of study participants (interviews-observations)

Row	Code	Qualitative research technique	Gender	Participant	Age (year)	Marital status	Level of education	History of participation in home care
1	A	Interview	Female	Nurse	51	Married	MS	20 years
2	B	Interview	Male	Nurse	36	Married	MS	10 years
3	C	Interview	Female	Nurse	32	Single	BS	8 years
4	D	Interview	Female	Nurse	37	Single	BS	7 years
5	E	Interview	Male	Nurse	37	Married	BS	9 years
6	H	Interview	Male	Nurse(supervisor)	45	Married	BS	12 years
7	N	Interview	Male	Nurse	40	Married	BS	5 years
8	Q	Interview	Female	Nurse(supervisor)	48	Married	MS	20 years
9	R	Interview	Female	Home care inspector	42	Married	BS	12 years
10	S	Interview	Male	Nurse	26	Single	BS	2 years
11	T	Interview	Female	Nursing assistant	32	Single	BS- Nursing assistance course	6 years
12	U	Interview	Male	Nursing assistant	28	Married	BS- Nursing assistance course	2 years
13	F	Interview	Male	Patient's son	41	Married	MS	3 months
14	O	Interview	Male	Patient's spouse	39	Single	BS	3 years
15	P	Interview	Female	Patient's daughter	35	Single	High school diploma	1 year and two months
16	W	Interview	Male	Home care physician	51	Married	Anesthesiologist and special care specialist	8 years
17	G	Observation	Female	Nurse	32	Married	BS	6 years
			Female	Patient's spouse	55	Married	High school diploma	6 months
			Male	Patient (ALS)	69	Married	MS	6 months
18	I	Observation	Female	Nurse	47	Single	BS	10 years
			Male	Patient's spouse	79	Married	High school diploma	9 months
			Female	Patient (CVA)	76	Married	MS	9 months
19	J	Observation	Male	Nurse	37	Married	BS	11 years
			Male	Patient's son	51	Married	MS	1 months
			Female	Patient (Lung Cancer)	86	Married	High school diploma	1 months
20	M	Observation	Male	Nurse	45	Single	MS	10 years
			Female	Patient's daughter	41	Married	MS	1 year
			Male	Patient (DM)	79	Married	MS	1 year

*MS* Master of Science, *BS* Bachelor of Science, *ALS* Amyotrophic lateral sclerosis, *CVA* Cerebral Vascular Accident, *DM* Diabetes Mellitus

### Data collection method

#### Interview

After receiving a letter of introduction, the researcher visited the home care centers of Tehran. The first participant was a nurse with 20 years of home care experience that was selected using purposeful sampling. Semi-structured interviews lasted about 75 min. The time and place of the interviews were determined by the participants. Prior to the interviews, written informed consent was obtained from the interviewees and the interviews were recorded with permission. Field notes were also used to record observations, events and mental ideas of the researcher. The interviews usually started with asking the participants about their home care experiences. Then, detailed questions were asked according to the topics raised in the previous interviews. In addition, an interview guide was used. Some of the interview guide questions for nurses, nurse assistants and family caregivers were as follows, "Based on your experience, what risks and problems have threatened your patient home care?", "What have you done to reduce these risks?", and "What measures did you take when your patient was in danger?" Probing questions were also used according to the participants’ answers and clarifying questions in the interview process in order to cover the research objectives. Finally, the participants were asked if there could think of any items that were not discussed. If necessary, supplementary questions were asked. It should be noted that the interviews were conducted in Persian. The quotes of the interviewees were translated from Persian to English by an expert. Then it was translated into Persian again to ensure no change in their original meaning. Two participants were asked to comment on whether the meaning of the quotations changed after re-translation and they approved the translation.

#### Observation

Since the focus of the grounded theory is to discover social processes and interactions, [33] the method of “observer as participant” was used to attract interaction with the participants. The researcher answered the patients' questions, trained the patients and family caregivers, monitored vital signs, and helped with moving and transferring patients. During the observations, the researcher asked any question that occurred to him in an open-ended manner and took notes at an appropriate time [34]. Observation sessions lasted for 8 to 10 h or even longer in patients' homes during a full shift (average: 9 h). In each observation session, all of the important safe care-related events, including the manner of providing care, supervisor and physician visits, nursing handover and family participation in care, and the researcher's interpretations and observations were recorded.

### Data analysis

Each interview or observation was immediately transcribed, coded and classified. After coding, if it was necessary to ask more questions from an interviewee, a supplementary interview was scheduled. Memo writing was also used during the coding process to determine the relationship between codes and concepts as well as the relationship between the codes resulting from the interviews and observations [35]. Data analysis was performed based on the approach proposed by Corbin & Strauss in 2015 [32] after transcribing the interviews and observations manually using the Comment option in the Microsoft Word software. This approach has several steps, including analyzing the data for concepts, elaborating the analysis, analyzing the process, and integrating concepts around a core category [32] (Table 2). The interview transcripts and observations were read several times. The main sentences were then coded based on the participants' statements. The codes were grouped based on their similar concepts. Then, the codes and the first classification were compared and similar items were merged. The strategies used for analyzing the data of the present study (all stages) included constant comparison (continuous interplay between analysis and data collection) and theoretical sensitivity (raised by memoing, the process of recording thoughts, feelings, decisions, ideas, processes and analytical insights as they emerge during the data collection, coding, analysis process and Previous experience of the researcher in caring for patients at home). we used a constant comparative method whereby particular data points were constantly compared to other data points in order to form categories and concepts [32]. After focusing on the conditions and the context in which the phenomenon occurred as well as factors that control the phenomenon, the core variable was determined [32].

**Table 2:** Analysis steps using the grounded theory method and Corbin & Strauss's approach [32]

Stages	Description (Action)
1. Analyzing the data for concepts	In this stage, the interviews and observations were transcribed by the researcher (S.K.) within 24 h after each interview to maintain the data integrity. The transcripts were analyzed using the Comment option in the Microsoft Word software with active participation of the research team. Then, codes were assigned to the transcripts. In fact, the data were broken into the smallest possible components. Codes that implied the same meaning and concept were placed in a single conceptual category. In this stage, note-taking, constant comparison, field note and theoretical sampling were used as part of the analysis process
2. Elaborating the analysis	In this stage, events that were conceptually similar to previously coded events were placed in the same category. Each new event that was placed in the same category added to its general characteristics and dimensions and enriched is diversity. Then, the concepts were placed in more abstract categories according to their characteristics and dimensions. Here, the researcher constantly used the constant comparison method and compared different parts of an interview with other parts, as well as the codes and categories resulting from one interview with other interviews. This process continued until reaching a complete description of each category in terms of its dimensions and characteristics. All members of the research team commented on and discussed the emerging codes and categories in weekly meetings
3. Analyzing data for context	In this stage, the researchers identified different conditions in which the studied phenomenon occurred. They also considered the conditions affecting the phenomenon and identified the situations that led to emergence of a safe care model. The structural/contextual factors affecting the process were also identified
4. Bring process into the analysis	In this stage, the researchers investigated the participants' strategies and behaviors. They further immersed themselves in the categories while simultaneously performing constant comparison. They sought to discover the process of patient safety in home health care as the main theme
5. Integrating concepts around a core category	In this stage, the main category was identified. Besides, the subcategories of the main category were identified and a theory was developed. Notes were reread, story lines were created, and diagrams were drawn to achieve final integration. In addition, a reflective coding matrix was used to describe the main category more precisely and determine its characteristics, dimensions, process, context and outcomes. Data saturation occurred when no new codes were identified and categories were coherent and logically relevant

### Trustworthiness and rigor

The four-dimension criteria, including credibility, dependability, confirmability and transferability [36], were used to evaluate the quality of qualitative studies [36]. In order to achieve credibility, interview transcripts, observations and codes were analyzed by grounded theory experts. Persistent observation, prolonged engagement in the data collection process, supplementary interviews, member checking, data triangulation and control of categories and themes by participants (member check) were other strategies used to ensure credibility. To confirm the dependability of the findings, the researcher transcribed the interviews and observations as soon as possible and sought help from two external reviewers using the auditing technique. To ensure transferability, field notes were used to fully describe the field under study at different stages of the study. To achieve confirmability, the researcher was fully aware his presuppositions and biases and limited subjectivity.

## Results

**Fig. 1:**
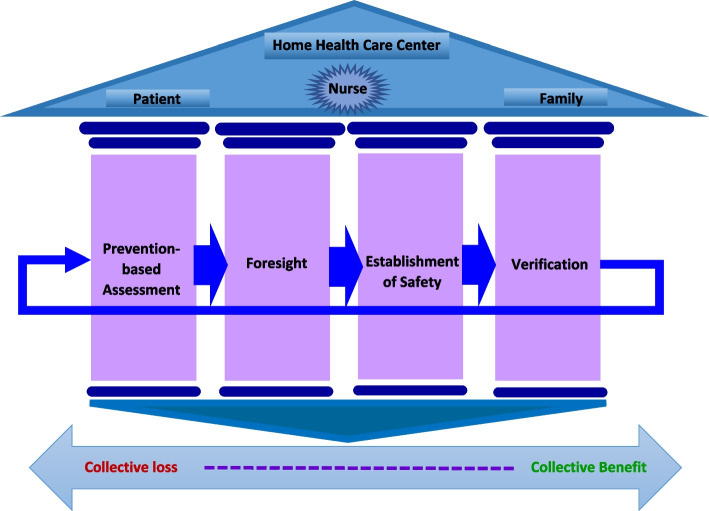
Model of patient safety in home health care

Considering the main categories extracted from the data using the grounded theory approach, the model of patient safety in home health care consisted of four stages, including assessment based on prevention, foresight, safety and verification (Table 3). The results also showed that patient safety in home health care was a model, which would bring benefits to the healthcare team if implemented properly by the nurse, family (family caregiver), patient and Home Health Care center (Fig. 1). Based on this, healthcare team involved in home care implemented strategies in order to maintain the model of safe patient care at home. These team first implemented a Prevention-based assessment strategy to identify risks. Then, in the second stage, they Foresight problems and solved them. In the third stage, they tried to establish safety in the implementation of their care, and in the final stage, they verified what they have done so far to ensure safe care.

**Table 3:** Categories, sub-categories and some statements of the participants in the model of patient safety in home health care

Main category	Sub-category	Primary categories
Prevention-based assessment	Primary assessment	Examining the requirements for safe patient transfer to home (first entry)
		Assessment during patient handover to next shift
		Assessment for self-protection
	Secondary assessment	Comprehensive patient examination
		Checking all connections
		History taking
		Checking the equipment and facilities
		Checking the drug storage
		Checking the patient environment and position
	Dynamic assessment	Continuous assessment of the patient's condition
		Continuous assessment of the patient's medication and equipment function
Foresight	Predicting problems	Estimating the possible risks and problems
		Predicting and estimating the deficiencies
	Organization and supply (facilities)	Eliminating deficiencies
		Organizing the environment around the patient
Establishment of safety	Environmental safety	Redesigning the environment
		Adaptation to the environment
	Targeted safety	Safe nutrition
		Providing safe personal hygiene
		Controlling infection
		Controlling immobility complications
		Airway protection
		Establishing safe activity and relaxation
	Safety during emergencies	Basic resuscitation safety
		Quick patient transfer to the hospital
	Medication safety	Adhering to the principles of prescribing emergency drugs (consulting a doctor or supervisor over the phone)
		Adhering to the principles of prescribing common medicines
	Remote/ participatory safety	Safety by educating the family
		Safety by delegation
		Safety by reassuring and building trust
		Direct consultation
		Remote consultation
Verification	Assessment	Gaining confidence through assessment
		Gaining confidence by taking and presenting reports
	Monitoring and control	Direct supervision
		Remote supervision
		Direct reporting
		Remote reporting

### Categories and sub-categories

#### Prevention-based assessment

The first step in patient safety in home health care was prevention-based assessment. The healthcare team tried to evaluate the patient's condition, position, medicines, environment, and equipment based on a preventive approach upon arriving home in order assess the important cases and determine the possible risks to implement safe care. Prevention-based assessment was conducted in three stages, including primary assessment, secondary assessment, and dynamic assessment.

##### Primary assessment

This assessment was performed upon arriving at home or handing the patient over as the first step. All the necessary items for the safe transfer of the patient or the continuation of the safe patient care were assessed by all the healthcare team members based on a preventive approach.

"I always go to the patient's home before the patient, and I check all the equipment and supplies previously prepared by the medical team at home." (Nurse Supervisor H)

"During patient handover, I first assess the patient to see if there are any problems so that hey do not pose a danger to them later." (Nurse B)

"The first time my patient wanted to go home, as the center supervisor had told me, I started to check whether all the items of the checklist were prepared, for example, I checked the cupboard." (Patient F's spouse)

##### Secondary assessment

Secondary assessment was a type of comprehensive assessment that was performed by the nurse on patients, connections, history and records, equipment and facilities, drug storage inventory and the patient environment and position.

"After arriving at the patient’s house, I first check my patient from head to toe." (Nurse A)

"The very first time I start evaluation, I even check the position of my patient's bed, for example to make sure it is not in the middle of the living room." (Nurse C)

"I check the patient's ventilator and oxygen concentrator to make sure it's working because their breathing depends on it." (Nurse D)

"After checking the oxygen concentrator, the nurse took the patient's medicine card, went to the patient's medicine cabinet and checked the drug storage for the next 24 hours" (Observation I)

##### Dynamic assessment

The nature of the home environment is very unpredictable; therefore, to prevent these conditions, the nurses performed the assessment process regularly throughout their shift. For example, they repeatedly checked the patient’s condition, medication, and equipment.

"While the nurse is sitting writing down her/his report, (s)he is looking at the patient's monitor at the same time, and is constantly evaluating the patient’s condition. Sometimes the nurse stands up and touches the oxygen concentrator to see if it is hot or not." (Observation G)

“Well, the patient's consciousness is something that we are constantly evaluating during a 24-hour period. We will definitely check their consciousness and their oxygen capsule constantly." (Nurse E)

#### Foresight

The healthcare team always paid some kind of attention to the future in order to maintain a safe model. The home foresight strategy was necessary due to the risky and unpredictable environment of the home. Predicting problems, organizing, and providing predictive facilities were among the main measures in foresight strategy.

##### Problem prediction

To predict the problems in the home environment, the healthcare team first estimated the possible risks and problems. Then, they predicted and estimated the existing deficiencies as well as the strategies required to eliminate those threats.

"A really good nurse who can predict must be able to predict the problem before it occurs." (Nurse B)

"One is I saw that a patient with tracheostomy did not have an Ambu bag. I predicted what would occur if he had a breathing problem, so I quickly called the center to prepare it." (Nurse A)

##### Organization and provision of equipment

Depending on the perceived threat, equipment or medicines were sometimes needed to be available at home in case a problem occurred. In some cases, the perceived threat was a kind of untidiness around the patient that had to be resolved.

"I always keep these drawers of the patient's supplies and medicines in their right place so that if there is a problem, I can find it as soon as possible." (Nurse D)

"...the nurse turned to the supervisor and said, “I think the patient may have shortness of breath. Can you get me a salbutamol spray? Because I found that there is not any.”" (Observation J)

"The nurse asked the patient's son to move his father's bed, which was attached to the wall, to the middle of the room so that the nurse could easily move around the patient in case a problem occurred." (Observation J)

#### Establishment of safety

##### Environmental safety

Since the home environment is not designed for caring, the patients constantly face many risks. Since the home environment is mostly small (apartments), changes in the standard care are very unlikely. If the home condition was not favorable, the healthcare team redesigned the environment. Otherwise, they coped with the limited environment and changed the conditions as far as the environment allowed.

"Once I went to the patient's home. I saw that the patient’s bed was at the kitchen door, I asked the family to vacate a room to relocate the patient." (Nurse E)

"When the window was open, it was above my father's head and could have hurt him, so I bought sliding windows." (Patient F' son)

##### Targeted safety

Establishment of targeted safety means implementing some measures to achieve specific safety goals. Safe nutrition, personal hygiene, controlling infection and immobility complications, establishing safe activity and relaxation, and protecting the airway were among these targeted measures.

"Prior to each meal, the nurse checked the patient's vital signs and suctioned the patient's mouth for excessive secretions. After that, (s)he asked the family to raise the bed head to 30°." (Observation I)

"The nurse told the family that due to the unstable condition of the patient, it is not possible to transfer him/her to the bathroom, and they should bathe the patient in bed". (Observation G)

"I always ask the nurses to disinfect their hands when they enter our home." (Patient G's spouse)

"I always help the nurses to change my father's position on time. I am very careful that he doesn't get a bed sore. Sometimes, when he is better, we help him to sit in a chair with the nurse's permission." (Patient F's son)

##### Safety during emergencies

In order to establish safety in emergency situations or patient resuscitation in a targeted manner, the healthcare team began to implement safety upon resuscitation and, if necessary, transferred the patient to the hospital quickly.

"Suddenly the patient developed bradycardia and cardiac arrest. I immediately told the family to call 911. I told the patient's son to perform heart massage so that I could administer the drugs." (Nurse B)

"We noticed that the patient had shortness of breath. The nurse immediately asked the family to call 911 and started Ambu ventilation." (Observation J)

##### Medication safety

The nurses tried to use the safe medication principles (based on the eight rights of medication administration) at home. If needed, they consulted a doctor or supervisor over the phone for a new medication.

"I always consult with the doctor or supervisor over the phone or in person, depending on the circumstances, to start a new medication for my patient." (Nurse E)

"Home medication is no different from the hospital medication; we must follow exactly the same eight medicine administration rights." (Nurse E)

##### Participatory/remote safety

Home care nurses are alone and do not benefit from the presence of colleagues or doctors. In addition, the family usually does not employ a nurse due to its high cost. Therefore, the nurse hands over some tasks to the family and trains them on some care tasks such as changing the patient's position or resuscitation in emergency situations. Furthermore, if needed, the nurse also uses the advice and guidance of a doctor or supervisor remotely over the phone or virtually (WhatsApp, Telegram, video call, etc.).

"If the supervisor is home and I need advice, I ask him/her in person. If it's midnight, for example, I would call him/her." (Nurse B)

"While the nurse turned the patient to her side, the patient's spouse spread the patient's sheets on the other side and straightened them completely." (Observation G)

#### Verification

After the implementation of the above processes, to ensure that the safe patient care model was implemented correctly, the healthcare team confirmed the above steps through assessment and monitoring processes.

##### Assessment

At this stage, the healthcare team checked their previously taken measures to ensure safety during the implementation phase. They also ensured safe care implementation by taking or presenting a report in some cases. For example, the nurses wrote daily nursing reports or recorded their documents accurately during higher-level periodic visits.

"I always reassess the patient and the condition after doing my tasks and in my free time to make sure my patient is safe." (Nurse E)

"The nurse began reassessing the patient, vital signs, connections, and equipment before the next nurse entered the home." (Observation G)

##### Monitoring and control

The nurse is alone at home and can use the help of other colleagues when a supervisor or doctor is present at home for an in-person visit. In this case, the nurse benefits from direct supervision and direct reporting to him/her. Otherwise, it uses indirect monitoring and reporting, which is usually performed by sharing the patient's condition through phone calls, video calls, or sharing patient photos or videos in online groups.

"The supervisor always makes periodic home visits and asks for reports during these visits. We also try to give him/her a complete report of what happened.” (Nurse C)

"Well, if the supervisor comes with the doctor for a visit, we will report to them and ask for their advice." (Nurse E)

"If the patient is in an acute condition and there is no resuscitation equipment, I will quickly call the supervisor or the doctor and report the condition to them, but if, for example, a patient's test is normal, I will take a picture and send it in the WhatsApp group." (Nurse A)

"The supervisor entered the home for a home visit at a due hour. He first talked with the family, and then went to the patient's room to monitor the patient, equipment, and medications." (Observation I)

### Collective benefit, collective loss

The outcomes of patient safety in home health care included two general aspects: Collective benefit and collective loss. In this study, based on the words of the participants, the result of the correct and step-by-step implementation of the process of safe patient care is collective benefit to all those involved in care, including: patient (patient safety, less injury, healing and saving lives, maintaining confidentiality and peace), nurse (no legal consequences, peace of mind, safety of the nurse), family (family security and peace of mind and family learning) and home care center (no legal consequences, security of the home care center).

Also, it was seen that if the process of safe care of the patient at home was not implemented properly, it would cause a collective loss for all those involved in the care, including: nurse (nurse concern, increase in complaints), family (family concern and cost imposition), patient (threat to the patient's safety, injury to the patient) and home care center (increase in complaints and damage and equipment malfunction).

### Foresight-based care

In this study, after discovering the concepts, main category and relationships between the categories, in order to create unity and coherence between the findings, the main category, which was repeated more than other concepts and connected other concepts and categories, was discovered. In this study, "foresight-based care" was found to be the main category and the basic process that connected other concepts and, at the same time, included all the categories. It seems that foresight flew in all categories and connected them like a string. The reason for the current perspective in home care is the different nature of care in an environment for which it is not designed and organized.

### Dangerous and unpredictable environment of home

In this study, the main concern of the participants was the concern about the unpredictable environment of home and its possible risks and dangers. The dangerous and unpredictable environment of the home created many risks for the patient's safety; therefore, all efforts and measures were aimed at identifying these safety threats for the patient. Hence, the concept of "prevention-based assessment" was the most important concept that affected the behaviors and functions of the members involved in home care in relation to safe patient care.

## Discussion

This was the first qualitative study that explained the model of patient safety in home health care. The results showed that assessment played a preventive role in most stages of safe patient care (primary, secondary, and dynamic). Some studies found that assessment was very important in planning and selecting preventive nursing measures when providing patient home care [37]. Evidence suggests that the position of detailed and general assessment of the records and facilities available at home should be considered because it is very helpful for preventing future risks and problems [38]. Dynamic assessment was investigated in the present study, which means continuous assessment of conditions and situations of home care to prevent risks. According to the evidence, patient assessment should be carried out not only at the beginning, but also periodically at all stages of patient care in order to prevent the possible causes of the patient safety compromise at home [39].

The present study found that foresight played a very important role in estimating and addressing possible risks. According to previous studies, prediction and elimination of risks is one of the main principles of home care due to its unstable conditions [40]. In addition, risk prediction helps to control the occurrence of serious dangerous events and increase the home care safety [41, 42]. Furthermore, provision of basic resources and equipment based on the previous predictions can play a key role in strengthening home safe care and services [9]. The available evidence supports the findings of the present study.

Our study also showed that safety should be maintained in all home care activities. High-quality, safe care has always been an integral part of nursing care [30]. The present study also found that the healthcare team, including the nurses, observed patient safety issues and made every effort to provide a safe model to achieve specific care goals in a targeted manner. According to the literature, nurses have always solved problems in a targeted manner by focusing on them and using detailed plans and effective measures [43]. One of these targeted cases is crisis management during home emergencies. A number of studies have discussed that the ability to manage acute conditions such as home resuscitation by a nurse and the correct reaction to it can imply safe care [44]. Another targeted safety case is medication safety. Considering the home conditions, despite the absence of other patients, medication errors or side effects may still occur [45]. Therefore, in order to provide safe medication during home care, nurses should always perform drug therapy in the form of a correct and high-quality process [45]. With regard to participatory/remote safety, the present study found that the nurse delegated some care measures to the patient's family members due to factors such as cost reduction.

Evidence shows that safety is a participtory process in all types of care [46]. To ensure the safety of proper home care, the participation and cooperation of the care team, i.e., the nurse, patient and family members, is needed [46]. The nurse uses training strategy to use this cooperation. According to some studies, nurses play an important role in implementing safe care through family training [47]. However, as shown in the present study and some other studies [48], inexperienced family members can threaten the patient's safety, and the nurse can resolve this problem through training. The present study also showed that continuous safety assessment, monitoring, and control were needed for verification. According to the Bener’s theory, in order to achieve the effectiveness of care and services and to ensure the proper delivery of these services, the assessment of one's own performance in the care process should be emphasized [49]. Evidence also suggests that the nurses should constantly monitor, record, and report all of their actions and activities to ensure their correct and safe implementation [50]. Accurate recording and timely reporting are important factors in preventing risks and improving patient safety [20, 51]. Due to the restrictions of the home care, nurses perform their home report writing and reporting online or via phone more frequently. The use of online technology, video call, or phone call can be a suitable solution to fill the distance gap [52]. Therefore, it can be stated that in order to monitor and control the conditions, if face to face attendance is not possible, it is possible to guarantee the patient's safety by using technology and remote care to provide services at home. The main category of the present study was foresight-based care, which was implemented by all members of the healthcare team (the nurse, center, family caregiver, and patient). The results of other studies have also revealed that apart from the nurse, other family members and the health care center are also involved in the model of patient safety in home health care [53]. According to the present study and other studies [24], foresight-based care not only involves safe patient care, but also benefits all healthcare team. Therefore, in order for home services and care to be safe for all members, including the patient, there is a need for serious cooperation and participation of the elements of care, i.e., the patient, the professional care provider, the family, and the officials of the center.

### Limitations

One of the limitations of the present study was the difficulty in inviting the participants, which was overcome through necessary arrangements by the attending home service centers and finding the names and addresses of the patients. Non-cooperation of some nurses, individuals and family members was also regarded as another limitation. The researcher collected their opinions by explaining the goals and the general process of the study.

## Conclusion

In the present study, in addition to achieving the general goal and determining the process of patient safety in home health care, foresight-based care was reported as the main category. The present study showed that the healthcare team played an important role in creating and promoting safe home patient care. It is essential to address individual risk assessments along with care assessment and support plans to adjust home care plans. Home care should focus on the risks that cause injury. It is also necessary to identify factors that can contribute to reducing and controlling these risks. The findings of the present study can be used to plan and implement applied clinical research in the future. It is also recommended to formulate practical guidelines and implement the model resulting from the present study in home health care services.

## Data Availability

The datasets generated and/or analyzed in the present study are not publicly available to ensure the privacy of the interviewed stakeholders; however, the data files are available in Persian from the corresponding author on reasonable request.
